# Draft Genome Sequence of *Loktanella cinnabarina* LL-001^T^, Isolated from Deep-Sea Floor Sediment

**DOI:** 10.1128/genomeA.00927-13

**Published:** 2013-11-14

**Authors:** Shinro Nishi, Taishi Tsubouchi, Yoshihiro Takaki, Ryo Koyanagi, Nori Satoh, Tadashi Maruyama, Yuji Hatada

**Affiliations:** Japan Agency for Marine-Earth Science and Technology, Yokosuka-shi, Kanagawa, Japana; Okinawa Institute of Science and Technology Graduate University, Onna-son, Okinawa, Japanb

## Abstract

This report describes the draft genome sequence of *Loktanella cinnabarina* LL-001^T^, which was the first isolated strain from deep-sea floor sediment of the genus *Loktanella*. The draft genome sequence contains 3,896,245 bp, with a G+C content of 66.7%.

## GENOME ANNOUNCEMENT

The genus *Loktanella*, which has been isolated so far from aqueous or marine environments, such as seawater, lakes, sea sand, marine biofilms, and microbial mats (1), belongs to the *Roseobacter* clade, the metabolic activity of which is suggested to play a crucial role in the global carbon cycle by converting dissolved organic carbon (DOC) into recalcitrant DOC (RDOC) in the ocean (2, 3). Recently, we isolated a novel strain, named *Loktanella cinnabarina* LL-001^T^ (1), and it is the first isolated strain from deep-sea floor sediment in the genus *Loktanella*. As the result of some biochemical tests, it was shown that strain LL-001^T^ has many carbohydrate-degrading capabilities (1). To reveal its characteristics in detail, we have performed draft genome sequencing, although the sequencing of two strains, *Loktanella hongkongensis* and *Loktanella vestfoldensis*, has already been done.

The draft genome sequencing of strain LL-001^T^ was performed on an Ion Torrent PGM sequencer (Life Technologies) (4) equipped with a 318 chip using 400-base chemistry. Data from the genomic DNA library contained 689,873 reads and 212,807,837 nucleotide bases, with an average read length of 308 bp. Assembly using the CLC Genomics Workbench version 6.01 (CLC bio Japan, Inc.) generated 192 contigs with a maximum contig size of 209,957 bp and an N_50_ contig length of 46,479 bp. The draft genome comprises 3,896,245 nucleotides with a G+C content of 66.7%. Gene prediction and annotation were determined by a combination of the Rapid Annotations using Subsystems Technology (RAST) 4.0 (5) pipeline, RNAmmer 1.2 (6), and tRNAscan-SE 1.23 (7). The genome of strain LL-001^T^ contains 3 rRNAs and 41 tRNAs. RAST analysis showed that the draft genome contains 3,802 predicted protein-coding sequences (CDSs), of which 78.0% (2,964) were annotated based on known proteins with biological functions and 22.0% (838) were annotated as hypothetical proteins.

Most of the protein-coding genes were related to metabolic traits, such as carbohydrate (12.8%), amino acids and derivative (6.7%), cofactor-vitamin-prosthetic group-pigment (6.3%), protein (6.0%), and membrane transport (4.2%). Genes encoding ABC transporters, which were responsible for the conversion of DOC into RDOC in the *Roseobacter* clade (3), were found in strain LL-001^T^, such as *xylFHG*, *frcABC*, *smoEFGK*, *aglEFGK*, *rbsABC*, *lolCDE*, and *lptBFG*. Some enzymes of interest for their uses in food processing and medical supplies were found, for example, β-galactosidase, α-galactosidase, 4-α-glucanotransferase, glucoamylase, glucosyltransferase, and lysozyme. The homologies with these known enzymes (homologies with non-*Loktanella* organisms) are 86% (72%), 80% (69%), 84% (62%), 75% (48%), 86% (62%), and 82% (48%), respectively.

### Nucleotide sequence accession numbers.

This genome shotgun project has been deposited at DDBJ/EMBL/GenBank under the accession no. BATB00000000. The 192 contigs have been deposited under the accession no. BATB01000001 to BATB01000192.
